# Non-Calcined Layer-Pillared Mn_0.5_Zn_0.5_ Bimetallic–Organic Framework as a Promising Electrocatalyst for Oxygen Evolution Reaction

**DOI:** 10.1021/acs.inorgchem.2c00542

**Published:** 2022-06-14

**Authors:** Reza Abazari, Ali Reza Amani-Ghadim, Alexandra M. Z. Slawin, Cameron L. Carpenter-Warren, Alexander M. Kirillov

**Affiliations:** † Department of Chemistry, Faculty of Science, University of Maragheh, Maragheh 55181-83111, Iran; ‡ Applied Chemistry Research Laboratory, Department of Chemistry, Faculty of Sciences, Azarbaijan Shahid Madani University, Tabriz 53751-71379, Iran; § EaStCHEM, School of Chemistry, University of St Andrews, St Andrews, Fife, Scotland KY16 9ST, U.K.; ∥ Centro de Química Estrutural, Institute of Molecular Sciences, Departamento de Engenharia Química, Instituto Superior Técnico, Universidade de Lisboa, Av. Rovisco Pais, Lisbon 1049-001, Portugal

## Abstract

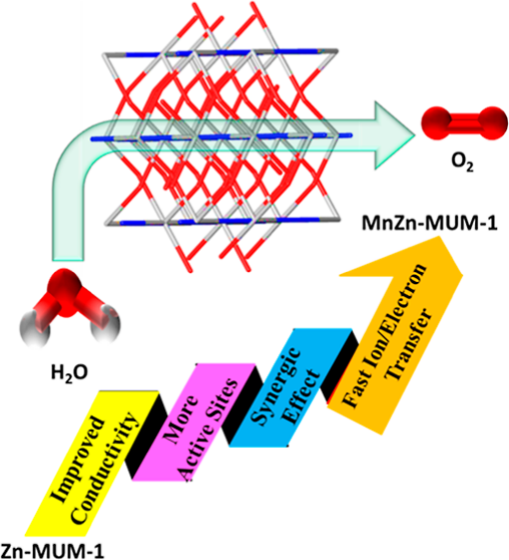

Electrocatalytic generation of oxygen is of great significance for sustainable, clean, and efficient energy production. Multiple electron transfer in oxygen evolution reaction (OER) and its slow kinetics represent a serious hedge for efficient water splitting, requiring the design and development of advanced electrocatalysts with porous structures, high surface areas, abundant electroactive sites, and low overpotentials. These requisites are common for metal–organic frameworks (MOFs) and derived materials that are promising electrocatalysts for OER. The present work reports on the synthesis and full characterization of a heteroleptic 3D MOF, [Zn_2_(μ_4_-odba)_2_(μ-bpdh)]_*n*_·*n*DMF (Zn-MUM-1), assembled from 4,4′-oxydibenzoic acid and 2,5-bis(4-pyridyl)-3,4-diaza-2,4-hexadiene (bpdh). Besides, a series of heterometallic MnZn-MUM-1 frameworks (abbreviated as Mn_0.5_Zn_0.5_-MUM-1, Mn_0.66_Zn_0.33_-MUM-1, and Mn_0.33_Zn_0.66_-MUM-1) was also prepared, characterized, and used for the fabrication of working electrodes based on Ni foam (NF), followed by their exploration in OER. These noble-metal-free and robust electrocatalysts are stable and do not require pyrolysis or calcination while exhibiting better electrocatalytic performance than the parent Zn-MUM-1/NF electrode. The experimental results show that the Mn_0.5_Zn_0.5_-MUM-1/NF electrocatalyst features the best OER activity with a low overpotential (253 mV at 10 mA cm^–2^) and Tafel slope (73 mV dec^–1^) as well as significant stability after 72 h or 6000 cycles. These excellent results are explained by a synergic effect of two different metals present in the Mn–Zn MOF as well as improved charge and ion transfer, conductivity, and stability characteristics. The present study thus widens the application of heterometallic MOFs as prospective and highly efficient electrocatalysts for OER.

## Abstract 2

New bimetallic MOF-based electrocatalysts were assembled, fully characterized, and explored in the oxygen evolution reaction, highlighting the impact of a second metal on the observed electrocatalytic activity.

## 1 Introduction

Oxygen evolution reaction (OER) is an important half-reaction for O_2_ generation in metal-air secondary batteries and water splitting.^1−3^ However, it is a kinetically limiting process for electrochemical systems owing to a multistep electron transfer (4OH^–^ → 2H_2_O + O_2_ + 4e^–^).^4^ Benchmark electrocatalysts for OER are based on IrO_2_ and RuO_2_, which are expensive and have limited use.^5−7^ Therefore, the design of advanced electrocatalysts for OER that rely on abundant and low-cost metals represents an important research topic. Despite many reports on inorganic OER electrocatalysts comprising transition-metal oxides, phosphides, carbides, sulfides, or different types of derived composites, there are issues of stability, low conductivity, and modest catalytic performance that require further improvements.^8−12^

As a group of porous crystalline materials, metal–organic frameworks (MOFs) have emerged as remarkably promising materials for photocatalysis, water splitting, supercapacitors, and Li-ion batteries owing to their unique features such as abundant active sites, high specific surface areas, and structural tunability.^13−17^ Many MOFs composed of inexpensive transition metals (e.g., Cu, Ni, Co, Fe, etc.) and carboxylate linkers are suitable candidates for advanced electrocatalytic systems.^18−21^ In some MOFs, metal nodes are coordinated by organic linkers as well as by solvent-based ligands; the latter are labile and can provide unsaturated metal centers.^22,23^ These can function as Lewis acid sites and act as electron acceptors, the features that are particularly important for the OER performance.^24,25^ Besides, the presence of active functional groups in organic linkers, such as amino groups, has a significant effect on electrocatalytic and electron transfer reactions.^26^ However, more research is needed to expand a typically low conductivity and insufficient stability of pristine MOFs while better understanding the mechanisms and roles of active sites.^27,28^ Guo and co-workers have briefly reviewed the progress that pristine MOFs have made in the field of electrocatalysts.^29^

There are a good number of recent studies on the use of heterometallic MOFs in electrocatalysis.^5,30^ For example, Xiong’s group reported that bimetallic Co–Fe MOFs can act as effective electrocatalysts due to the presence of two metals.^31^ An improvement in electrocatalytic behavior is usually imputed to synergic effect between different metals and also to better conductivity and stability.^32^ A good OER performance of Ni–Co MOF nanosheets was described by Tang and co-workers,^33^ while a related study on bimetallic Ni–Fe MOFs was carried out by Zheng et al.^34^ In another report, Duan and co-workers explored the electrocatalytic behavior of heterometallic MOFs for splitting of water.^35^ Lu et al. described an electrocatalytic performance of Fe_2_Ni-MOF/NF (NF = Ni foam) with a low overpotential of 240 mV at a current density of 10 mA cm^–2^.^36^ Fransaer et al. synthesized a Co–Ni MOF and investigated its OER activity.^37^ Dolgopolova’s group showed that by changing and engineering metal nodes in MOFs, their electronic properties can be modulated and improved.^38^ In many heterometallic MOFs, their topologies are unpredictable, and often the frameworks are fragile. However, in some MOFs, the parent structure is somewhat retained upon the introduction of the second metal.^39^ For example, Botas and co-workers showed that a small amount of Co^2+^ ions (less than 25%) can replace Zn^2+^ nodes in the original structure.^40^ A similar behavior was observed for ZIF-8/ZIF-67 and HKUST-1.^41−45^

In recent years, Mn-based MOFs have been widely surveyed as electrocatalysts for OER.^46^ The advantages of manganese(II) MOFs as electrocatalysts in comparison with other types of 3d-metal-based frameworks concern an increased number of active sites, which simplifies the diffusion of electrolyte ions and enhances the overall electrocatalytic performance.^47,48^ An interesting strategy concerns the replacement of Zn^2+^ nodes in a standard MOF structure with the Mn^2+^ nodes to create a heterometallic material.^49^

In the present study, we prepared a heteroleptic 3D MOF, [Zn_2_(μ_4_-odba)_2_(μ-bpdh)]_*n*_·*n*DMF (Zn-MUM-1), using 4,4′-oxydibenzoic acid (H_2_odba) and 2,5-bis(4-pyridyl)-3,4-diaza-2,4-hexadiene (bpdh) as linkers. This Zn(II) MOF was applied as a model structure for incorporating the second metal, namely, manganese(II). As a result, a series of heterometallic MnZn-MUM-1 frameworks (abbreviated as Mn_0.5_Zn_0.5_-MUM-1, Mn_0.66_Zn_0.33_-MUM-1, and Mn_0.33_Zn_0.66_-MUM-1) was also assembled, characterized, and used for the fabrication of working electrodes based on Ni foam (NF), followed by their exploration in OER. It should be mentioned that the reports on the electrocatalytic oxygen evolution systems that rely on layer-pillared bimetallic MOF materials are still scant. Therefore, the design of both homo- and heterometallic MOFs as electrocatalysts can open new perspectives in terms of interesting relationships between the structure and performance in OER.

## 2 Experimental Section

### 2.1 Synthesis of Zn-MUM-1

Zn-MUM-1 (MUM = Material from University of Maragheh) was prepared by the reaction of H_2_odba and bpdh with Zn(NO_3_)_2_·6H_2_O in dimethylformamide (DMF) solvent using the solvothermal method. H_2_odba was obtained from a commercial supplier, while a bpdh pillar was prepared according to a previously reported protocol with slight modifications.^50^ The synthesis of Zn-MUM-1 was performed using a single-step solvothermal method. Zn(NO_3_)_2_·6H_2_O (0.25 mmol), H_2_odba (0.25 mmol), and bpdh (0.15 mmol) were dissolved in DMF (6 mL, for each reagent) upon sonication (20 s). The obtained clear solution was transferred to a Teflon-lined stainless-steel vessel (10 mL volume), closed, and kept in an oven at 75 °C for 36 h. The vessel was then gradually cooled (10 °C/h) to room temperature and opened. The orange block crystals were washed with DMF (10 mL) at least three times under sonication to remove any excess of organic ligands. Finally, the product was dried under vacuum for 24 h to give microcrystalline Zn-MUM-1. Powder X-ray diffraction (PXRD) analysis confirmed the phase purity of the product. Yield: 76 mg (74% based on Zn). Single crystals of Zn-MUM-1 suitable for X-ray diffraction were withdrawn from the reaction solution before washing and drying procedures.

### 2.2 Synthesis of Heterometallic MnZn-MUM-1 Samples

For the synthesis of Mn_0.5_Zn_0.5_-MUM-1, a DMF solution (3 mL) of Zn(NO_3_)_2_·6H_2_O (0.125 mmol) and Mn(NO_3_)_2_·4H_2_O (0.125 mmol) and a DMF solution (3 mL) of H_2_odba (0.25 mmol) and bpdh (0.15 mmol) were prepared. Then, both solutions were mixed in a vial (10 mL) under vigorous stirring for 24 h at 60 °C. After cooling the reaction mixture to room temperature, DMF (10 mL) was added, and the obtained suspension was stirred for 30 min, followed by centrifugation. This washing process was repeated three times. Finally, the solid product was separated and dried under vacuum for 24 h to give Mn_0.5_Zn_0.5_-MUM-1. Yield: 29 mg. Samples of MnZn-MUM-1 with the Mn/Zn molar ratios of 1:1, 2:1, and 1:2 were obtained in a similar manner, resulting in Mn_0.5_Zn_0.5_-MUM-1, Mn_0.66_Zn_0.33_-MUM-1, and Mn_0.33_Zn_0.66_-MUM-1 materials.

### 2.3 Topological Analysis

To better understand an intricate crystal structure of Zn-MUM-1, we carried out its topological analysis by applying a concept of the underlying net.^51−54^ Such a simplified net was generated by contracting the μ_4_-odba^2–^ and μ-bpdh blocks to the corresponding centroids while maintaining their connectivity with zinc(II) nodes.

### 2.4 Electrocatalysis

An Origaflex device was employed to study the electrochemical performance of the obtained MOF samples embedded into NF electrodes. The electrochemical tests were carried out in a three-electrode system in KOH (2 M) electrolyte with the Ag/AgCl, graphite rod, and fabricated electrode serving as the reference, counter, and working electrodes, respectively. The working electrodes were assembled by depositing MOF samples on a piece of NF (1 cm^2^). In brief, an aqueous suspension (60 μL total volume) containing activated carbon (1 mg), polytetrafluoroethylene (PTFE, 60 wt %, 40 μL), and the MOF sample (4 mg) was prepared. Then, 60 μL of this suspension was deposited by a pipetor on the NF followed by drying in air. The obtained Zn-MUM-1/NF and MnZn-MUM-1/NF electrocatalysts were then utilized as working electrodes. The OER performance of the electrocatalysts was assessed with rate linear sweep voltammetry (LSV), chronopotentiometry, electrochemical impedance spectroscopy (EIS), and cyclic voltammetry (CV). In all these tests, the values of potentials were converted into RHE using the following equation (eq 1)
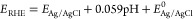
1

## 3 Results and Discussion

### 3.1 Structural Description

**Figure 1:**
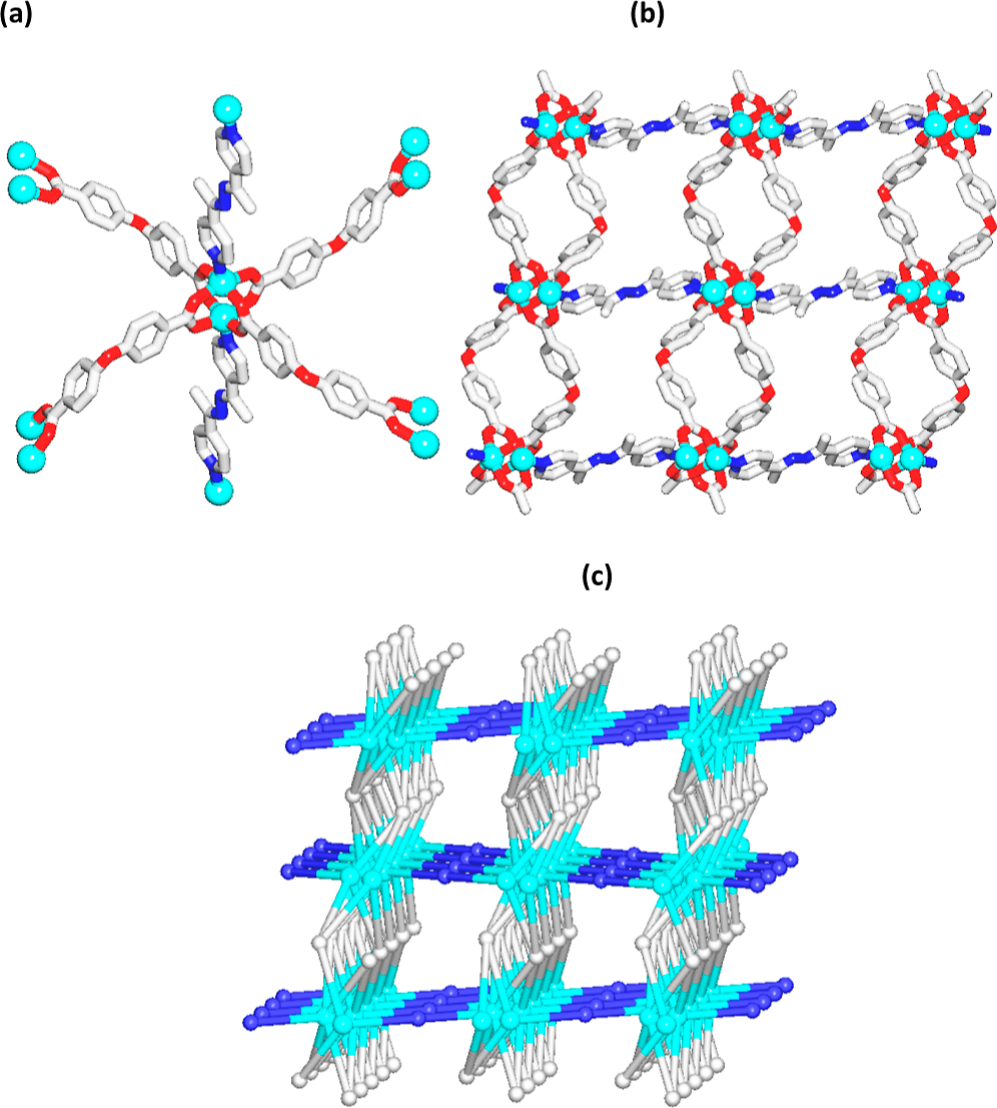
Fragments of the crystal structure of Zn-MUM-1. (a) Paddle-wheel dizinc(II) block and connectivity of μ_4_-odba^2–^ and μ-bpdh ligands. (b) 3D layer-pillared MOF. (c) Topological view of a binodal 4,5-linked net with a 4,5T50 topology. Further details: (a,b) H atoms and DMF solvent molecules were omitted for clarity, color codes: Zn (cyan), O (red), N (blue), and C (gray); (b,c) view along the *b* axis; (c) 5-connected Zn1 nodes (cyan), centroids of 4-connected μ_4_-odba^2–^ nodes (gray), and centroids of 2-connected μ-bpdh pillars (blue).

Single-crystal X-ray diffraction analysis of Zn-MUM-1 reveals a layer-pillared 3D MOF (Figures 1; S1–S3 and Tables S1, S2, Supporting Information). Per asymmetric unit, there is one Zn(II) center, one μ_4_-odba^2–^ dicarboxylate block, half of a μ-bpdh pillar, and one DMF solvent molecule. The Zn1 atom is five-coordinated and adopts a distorted square-pyramidal {ZnNO_4_} coordination geometry with the Zn–N and Zn–O distances in the 2.033(2)–2.050(2) Å range. The environment around the Zn1 atom is occupied by four carboxylate O donors from four μ_4_-odba^2–^ blocks in equatorial sites and one N donor from the μ-bpdh pillar in an axial position (Figure 1a). The COO^–^ groups of μ_4_-odba^2–^ feature a bridging bidentate mode. Four μ_4_-odba^2–^ blocks interconnect the two adjacent Zn1 atoms into paddle-wheel dizinc(II)-tetracarboxylate blocks with a short Zn1···Zn1 separation of 2.9442(5) Å. These are further cross-linked by the remaining carboxylate functionalities of μ_4_-odba^2–^ into 2D layer motifs. Finally, the adjacent layers are pillared into an intricate 3D MOF structure by means of the μ-bpdh linkers (Figure 1b). The solvated structure features some porosity with voids occupying 8.8% (446.7 Å^3^) of the unit cell volume, according to the analysis of voids by Mercury software and using a spherical probe (radius: 1.2 Å, grid spacing: 0.7 Å). Additional information about this structure is available in the Supporting Information file. Topological classification of the simplified net of Zn-MUM-1 reveals a binodal 4,5-linked framework that is built from the 5-linked Zn1 and 4-linked μ_4_-odba^2–^ nodes, in addition to the 2-connected μ-bpdh linkers (Figure 1c). This framework can be topologically classified within a 4,5T50 type and defined by a (4^2^.8^4^) (4^6^.10^4^) point symbol; herein, the (4^2^.8^4^) and (4^6^.10^4^) notations belong to the μ_4_-odba^2–^ and Zn1 nodes, respectively. Although this topology has been theoretically predicted, it has not yet been identified in isolated MOFs.

**Figure 2:**
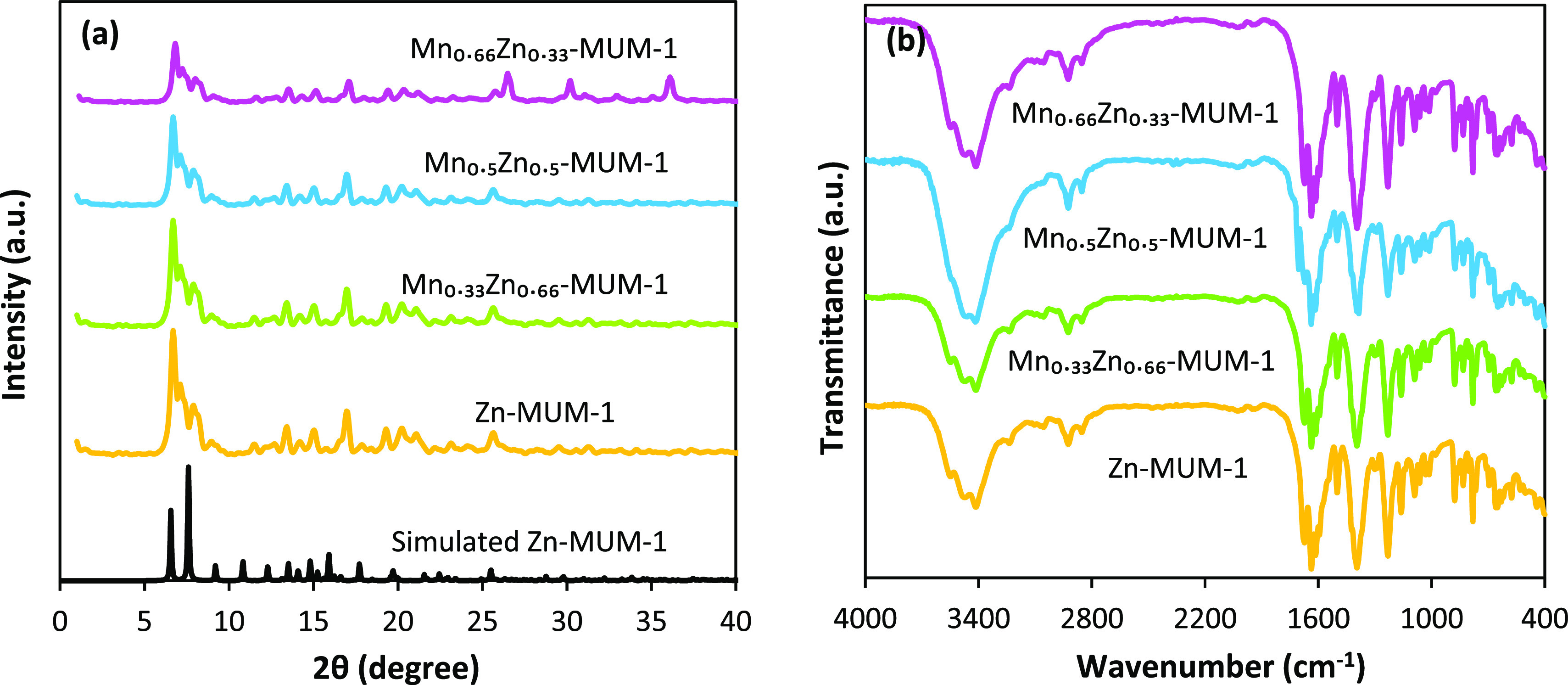
(a) PXRD patterns and (b) FT-IR spectra of Zn-MUM-1 and MnZn-MUM-1.

PXRD patterns of Zn-MUM-1, Mn_0.5_Zn_0.5_-MUM-1, and Mn_0.33_Zn_0.66_-MUM-1 are shown in Figure 2a, revealing the characteristic peaks at 2θ values of 11.29, 11.72, 15.06, 23.29, 28.58, and 34.51°, respectively. These are in a good agreement with the simulated pattern. The presence of sharp peaks indicates the high crystallinity of the samples, while a relative similarity of PXRD patterns of Zn-MUM-1, Mn_0.33_Zn_0.66_-MUM-1, and Mn_0.5_Zn_0.5_-MUM-1 suggests an incorporation of Mn ions without altering the parent MOF structure. However, the structural pattern of the Mn_0.66_Zn_0.33_-MUM-1 sample shown in Figure 2a undergoes a significant change. Such results were reported in other studies.^39−45^Figure 2b shows the FT-IR spectra of the as-prepared Zn-MUM-1 and MnZn-MUM-1, which show similar characteristic bands. These include ν_as_(CH) and ν_s_(CH) bands in the 2800–3000 cm^–1^ range as well as strong ν_as_(COO) and ν_s_(COO) vibrations of carboxylate groups at 1573 and 1359 cm^–1^, respectively.^55−57^ Other bands correspond to standard absorptions associated with aromatic rings and DMF solvent molecules.^58−61^

**Figure 3:**
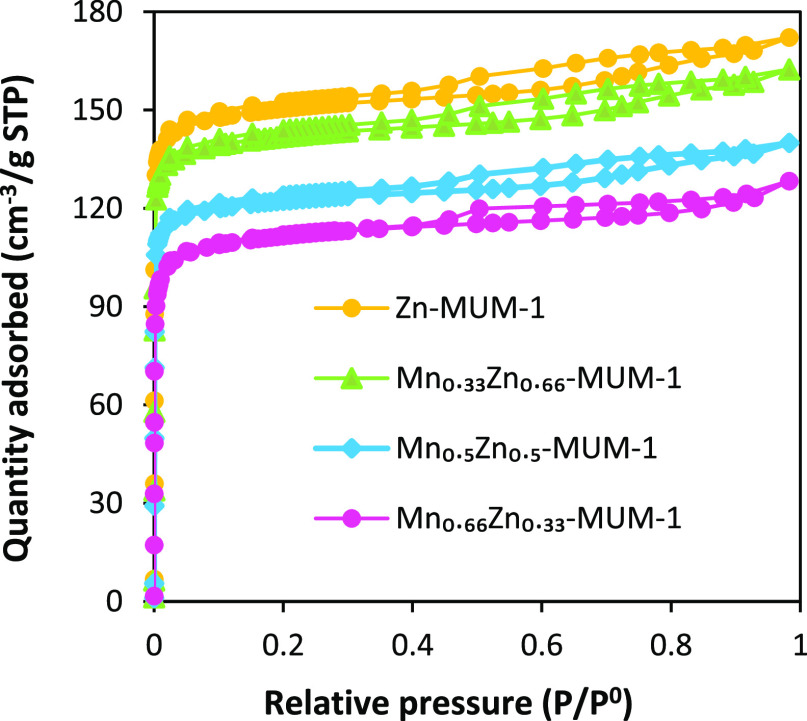
N_2_ adsorption–desorption isotherms collected at 77 K for the as-synthesized Zn-MUM-1 and MnZn-MUM-1 structures.

**Figure 4:**
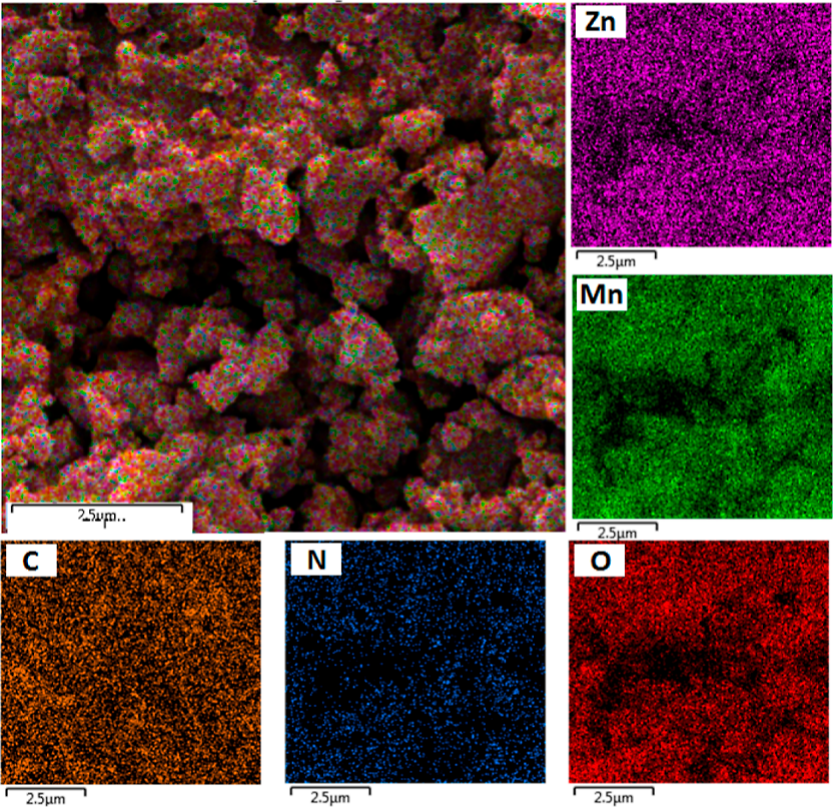
SEM image of Mn_0.5_Zn_0.5_-MUM-1 and the corresponding elemental mapping images for Zn, Mn, C, N, and O.

The porosity of the samples was evaluated by the N_2_ adsorption at 77 K (Figure 3). For Zn-MUM-1, a type I adsorption isotherm is observed with the BET surface area of 614 cm^2^/g and the pore volume of 0.572 cm^2^ g^–1^. Although the heterometallic samples reveal slightly smaller surface areas (511–585 cm^2^/g) than that of Zn-MUM-1, these values are still well acceptable for applications in electrocatalysis (Table S3, Supporting Information). Possible reasons for systematically lower BET surface areas of heterometallic samples are further discussed in the Supporting Information, along with relevant examples from literature. Also, the shape and type of the isotherms in heterometallic samples do not indicate a significant change if compared to Zn-MUM-1. Therefore, with high surface areas and microporous structures, MnZn-MUM-1 frameworks should be susceptible to OER activity owing to a facilitated transfer of electrolyte ions. The metal content in the obtained samples was determined by ICP-OES analysis (Figure S4, Supporting Information). As expected for the MnZn-MUM-1 structures, the amount of Mn increases upon augmenting its concentration in the starting reaction solution, but this increase is nonlinear and associated with different coordination abilities of metals.^62^ SEM/elemental mapping shows a homogeneous distribution of Zn, Mn, O, N, and C elements across the Mn_0.5_Zn_0.5_-MUM-1 surface (Figure 4). This experiment is particularly important to confirm a homogeneous embedding of Mn ions into a parent structure of Zn-MUM-1. These results are also in good agreement with the FT-IR and PXRD data. The FE-SEM images of the Mn_0.5_Zn_0.5_-MUM-1 at two different resolutions with a morphology of angular particles and flat surfaces are shown in Figure S5, Supporting Information.

**Figure 5:**
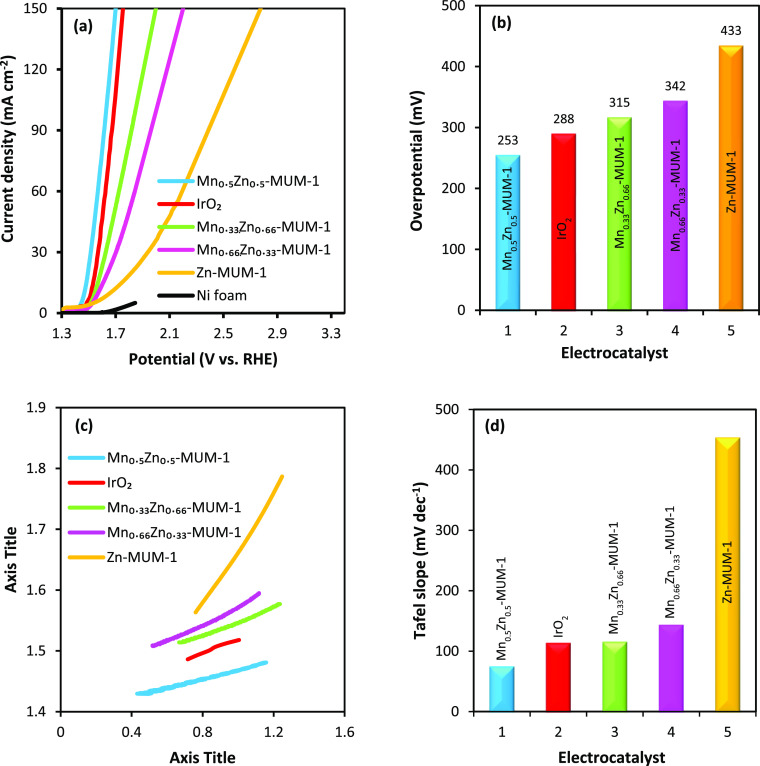
OER parameters for different electrocatalysts deposited on NF: (a) LSV curves at a 2 mV s^–1^ scan rate, (b) overpotential values at 10 mA cm^–2^, (c) Tafel plots, and (d) values of Tafel slopes.

To assess the OER performance of the obtained MOFs, these were incorporated into 1 cm^2^ pieces of NF to give the MnZn-MUM-1/NF working electrodes with different Mn-to-Zn ratios. Then, LSV tests were carried out in a three-electrode system in a 2 M KOH electrolyte and at a scan rate of 2 mV s^–1^ (Figure 5a). The Mn_0.5_Zn_0.5_-MUM-1/NF sample outperforms other electrocatalysts, even the commercial IrO_2_. Such a superior performance of the mentioned sample can be assigned to the (i) optimal ratio of the two metals with synergic effect, along with the presence of NF as a matrix and a source of the third metal, (ii) formation of a unique structure with open pores and channels, (iii) numerous electrochemically active metal centers, and (iv) improved conductivity that accelerates the electron and ion transfers and promotes the penetration of the electrolyte ions into the structure. Figure 5b shows the overpotentials of the tested electrocatalysts. The lowest overpotential (253 mV at a current density of 10 mA cm^–2^) was observed for Mn_0.5_Zn_0.5_-MUM-1/NF, while the overpotentials of IrO_2_/NF, Mn_0.33_Zn_0.66_-MUM-1/NF, Mn_0.66_Zn_0.33_-MUM-1/NF, and Zn-MUM-1/NF were 288, 315, 342, and 433 mV, respectively.

The performance of the samples was also kinetically assessed by the Tafel plots obtained from the LSV data (Figure 5c). As presented in the Tafel equation (eq 1), η shows the overpotential, β denotes the Tafel slope, *J* represents the current density, and *J*_0_ refers to the current density at zero overpotential (exchange current density). An efficient electrocatalyst with the best performance should possess the lowest Tafel slope. The Tafel slopes of Mn_0.5_Zn_0.5_-MUM-1/NF, IrO_2_/NF, Mn_0.66_Zn_0.33_-MUM-1/NF, Mn_0.33_Zn_0.66_-MUM-1/NF, and Zn-MUM-1/NF were 73, 112, 114, 142, and 452 mV dec^–1^, respectively (Figure 5d). From the kinetics perspective, the Mn_0.5_Zn_0.5_-MUM-1/NF electrocatalyst exhibits the best performance among all the tested samples, which can be attributed to faster penetration of electrolyte ions within the catalyst structure and faster electronic and ionic transport due to shorter paths as a result of the unique structure of the catalyst with open pores.^63^ The electrocatalysts were further evaluated by assessing another important parameter such as an electrochemically active surface area (ECSA). The ECSA is directly related to double-layer capacitance (*C*_dl_). Higher values of *C*_dl_ imply better electrocatalytic behavior. The ECSA was evaluated by CV in the non-Faraday current range at various scan rates. The *C*_dl_ values of Mn_0.5_Zn_0.5_-MUM-1/NF, Mn_0.33_Zn_0.66_-MUM-1/NF, Mn_0.66_Zn_0.33_-MUM-1/NF, and Zn-MUM-1/NF were 9.2, 5.8, 4.9, and 4 mF cm^–2^, respectively (Figure S6, Supporting Information). The highest *C*_dl_ value observed for Mn_0.5_Zn_0.5_-MUM-1/NF implies its better electrocatalytic performance, that is, the optimal metal ratio in this sample and its unique structure, which offer a major surface area for electrochemical reactions.

**Figure 6:**
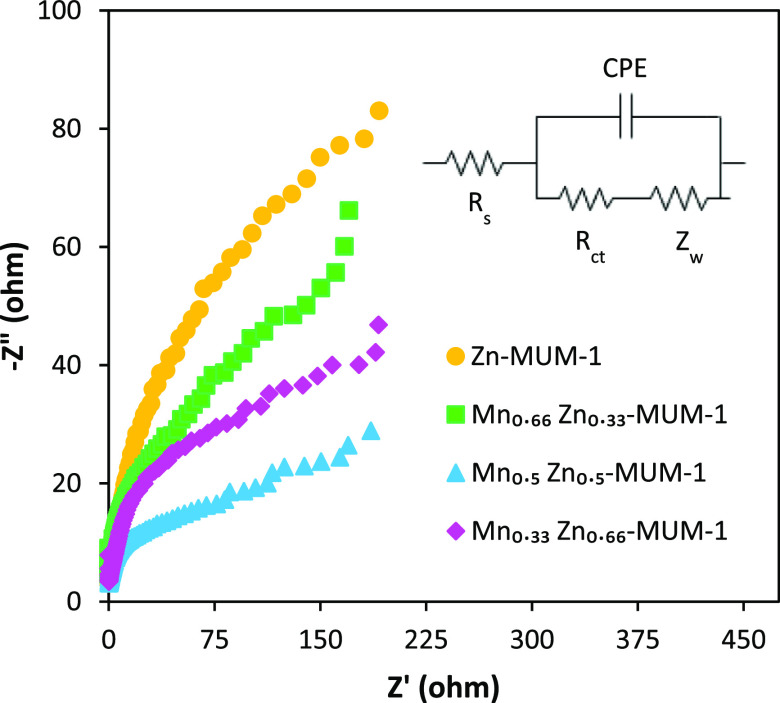
EIS results for different electrocatalysts deposited on NF (inset: equivalent circuit model).

**Figure 7:**
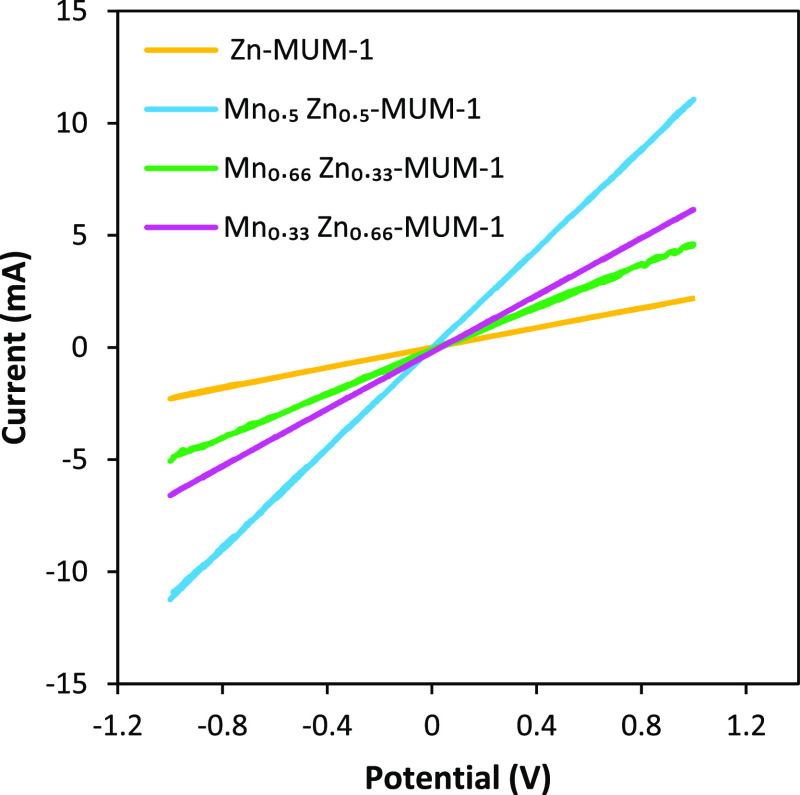
CV curves of the samples at a 100 mV s^–1^ scan rate to determine the conductivity.

The EIS spectra were also explored to investigate the kinetics of OER. According to the Nyquist plots (Figure 6), Mn_0.5_Zn_0.5_-MUM-1/NF exhibits the lowest charge-transfer value among the tested samples. A high surface porosity of this electrocatalyst accelerates ion and electron transport, thus improving its performance. After fitting the Nyquist plots data in Z-view software, the equivalent circuit model was also determined for these diagrams (inset of Figure 6). In the circuit model, *R*_s_, *R*_ct_, and *Z*_w_ are related to solution resistance, charge-transfer resistance, and Warburg resistance, respectively. Constant phase element (CPE) refers to the time constant that represents the surface porosity of the electrocatalyst. To determine the electrical conductivity, we prepared the pressed pills of samples from active material, PTFE, and activated carbon in 10:10:80 ratios. A CV test was performed at a 100 mV s^–1^ scan rate within a −1.0 to 1.0 V potential window. The result of this test was a linear dependence. According to the equation of *R* = *V*/*I*, with a higher line slope, the conductivity of the sample will be higher (Figure 7), which is observed for Mn_0.5_Zn_0.5_-MUM-1.

**Figure 8:**
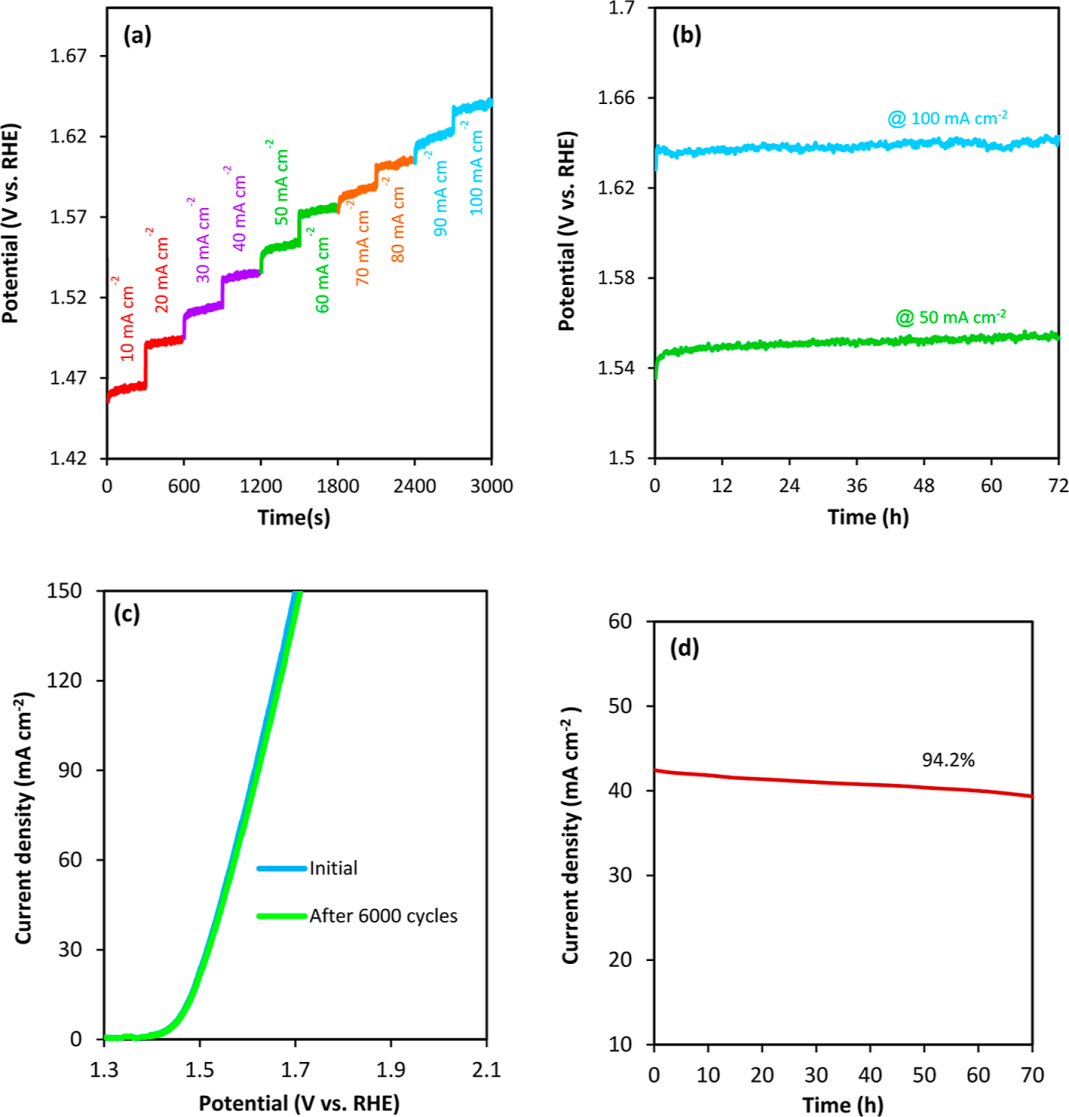
(a) Multistep chronopotentiometry plot at different current densities, (b) chronopotentiometry plots at current densities of 50 and 100 mA cm^–2^, (c) LSV curves of Mn_0.5_Zn_0.5_-MUM-1/NF before and after 6000 cycles, and (d) chronoamperometry curve at 1.53 V versus RHE for Mn_0.5_Zn_0.5_-MUM-1/NF.

One of the most important parameters in the performance of an electrocatalyst concerns its stability. Therefore, the stability of the most promising sample (Mn_0.5_Zn_0.5_-MUM-1/NF) was evaluated using different methods. Initially, the stability of the electrocatalyst was assessed at current densities of 10, 30, 50, 70, and 100 mA cm^–2^ using a multistep chronopotentiometry technique (Figure 8a). The potential was constant at each current density, but it immediately changed by altering the current density from 10 to 100 mA cm^–2^. The chronopotentiometry measurements were also performed for this electrocatalyst at 50 and 100 mA cm^–2^ for a longer period of 72 h (Figure 8b), revealing no significant changes in the overpotential of the electrode. Both these approaches confirm a high stability of this electrocatalyst. Yet another method to evaluate the stability consisted in repeating the LSV curve measurement after 6000 cycles. As shown in Figure 8c, no significant difference can be detected between the first curve and the one obtained after 6000 cycles, thus further confirming the high stability of this electrocatalyst. To confirm the stability of Mn_0.5_Zn_0.5_-MUM-1/NF at a potential of 1.53 V versus RHE, the chronoamperometric curve (*i*–*t*) was also recorded (Figure 8d), indicating that after 70 h, this electrocatalyst can maintain 94.2% of its initial current density. The long-term stability and excellent performance of Mn_0.5_Zn_0.5_-MUM-1/NF can be attributed to its unique structure with open pores and channels which accelerated electron and mass transfers, further facilitating the penetration of electrolyte ions into the cavities and channels. Therefore, the electrode contact with the electrolyte ions is enhanced, promoting the electrochemical reactions. Additionally, the synergic effect between different metals in the structure of the MOF and NF accelerated the electrochemical reactions and improved the conductivity and electrocatalytic performance.^34,64^

In general, the synergic effect refers to the positive influence from a combination of two or more components.^55^ The incorporation of a second metal could cause an increment in the electrocatalytic activity of the material as a result of altering its electronic and other properties. Porous bimetallic materials are promising electrodes in energy systems. Their porous structures offer high specific areas and facilitate the volume change, thus enhancing the reversible energy storage and cycling stability.^65^ In comparison with monometallic MOFs, the generation of a more complex heterometallic structure can result in the synergic coupling of components, which may further increase the advantages of each metal component, offering stronger redox activity, greater stability, faster charge/electron transfer rates, more controllable structures, and smaller band gaps due to various active sites with several oxidation states. Additionally, bimetallic structures can present superior electrochemical performance if compared to the monometallic materials, which can be assigned to the synergic effects of different metallic ions, enhancing the Faradaic reactions and boosting the electrical conductivity.^66^

## 4 Conclusions

In this work, we showed that a combination of two types of flexible building blocks with carboxylate and pyridine functionalities along with zinc(II) nodes can lead to the assembly of a heteroleptic 3D MOF (Zn-MUM-1). Apart from widening a growing family of functional MOFs, the present compound also contributes to the identification of metal–organic architectures with rare types of topologies. In addition, this MOF can be used as a structural model for designing heterometallic Mn(II)–Zn(II) derivatives that can maintain the structure of the parent MOF. Hence, a series of bimetallic MnZn-MUM-1 frameworks with different molar ratios between two metals (Mn_0.5_Zn_0.5_-MUM-1, Mn_0.66_Zn_0.33_-MUM-1, Mn_0.33_Zn_0.66_-MUM-1) was also assembled and investigated. Furthermore, all these frameworks were used as active components (electrocatalysts) for the fabrication of the corresponding working electrodes based on NF, followed by their exploration in the OER. The experimental results showed that the Mn_0.5_Zn_0.5_-MUM-1/NF material features a superior OER activity than that of the monometallic Zn-MUM-1/NF and other heterometallic MnZn-MUM-1/NF samples, also revealing a low overpotential (253 mV at 10 mA cm^–2^) and Tafel slope (73 mV dec^–1^) as well as significant stability after 72 h or 6000 cycles. These results are explained by the synergic effect of two different metals present in the MOF as well as improved charge and ion transfer, conductivity, and stability characteristics. The obtained data indicate that the regulation of the properties of MOFs by incorporation of the second electrochemically active metal in its structure represents a particularly promising path toward the design of efficient electrocatalysts.

## Supporting Information Available

Additional structural representations, crystal data and structure refinement details, and selected bond lengths and angles for Zn-MUM-1; ICP-OES analysis; FE-SEM images; CV curves; and N_2_ adsorption data (PDF)
