# Supplementary material for: Non-Calcined Layer-Pillared Mn0.5Zn0.5 Bimetallic–Organic Framework as a Promising Electrocatalyst for Oxygen Evolution Reaction
Source: Inorg Chem. 2022 Jun 14;61(25):9514–22. doi: 10.1021/acs.inorgchem.2c00542 (PMC9775468; doi:10.1021/acs.inorgchem.2c00542)
Supplement: Supplementary file 1 — ic2c00542_si_001.pdf [file ic2c00542_si_001.pdf]

Supporting Information (SI)

A Non-Calcined Layer-Pillared $Mn_{0.5}Zn_{0.5}$ Bimetallic–Organic Framework as a Promising Electrocatalyst for Oxygen Evolution Reaction

Reza Abazari,*,† Ali Reza Amani-Ghadim,*,‡ Alexandra M. Z. Slawin,§ Cameron L. Carpenter-Warren,§ and Alexander M. Kirillov*,⊥

†Department of Chemistry, Faculty of Science, University of Maragheh, P.O. Box 55181-83111, Maragheh, Iran, E-mail: reza.abazari@modares.ac.ir

‡Applied Chemistry Research Laboratory, Department of Chemistry, Faculty of Sciences, Azarbaijan Shahid Madani University, Tabriz 53751-71379, Iran, E-mail: amani.gh@azaruniv.ac.ir

§EaStCHEM, School of Chemistry, University of St Andrews, St Andrews, Fife, KY16 9ST, Scotland, UK

⊥Centro de Química Estrutural, Institute of Molecular Sciences, Departamento de Engenharia Química, Instituto Superior Técnico, Universidade de Lisboa, Av. Rovisco Pais, 1049-001 Lisbon, Portugal, E-mail: kirillov@tecnico.ulisboa.pt

---

General Methods. The N_2 adsorption/desorption isotherm was measured at liquid nitrogen temperature (77 K) by using a Micromeritics ASAP 2020 analyzer. The specific surface area was calculated by the Brunauer-Emmett-Teller (BET) method. A Thermo Nicolet IR 100 FT-IR instrument was employed to record the FT-IR spectra. The Powder X-ray diffraction (PXRD) patterns of the samples were recorded on a Philips X'pert diffractometer equipped with a Cu Kα radiation source. The inductively coupled plasma (ICP) analyses were performed on a Varian ICP-OES VISTA-PRO CCD instrument.

Single Crystal X-ray Diffraction. Single crystal X-ray diffraction data were collected at 125 K using a Rigaku MM-007HF High Brilliance RA generator/confocal optics with XtaLAB P200 diffractometer [Cu Kα radiation (λ = 1.54187 Å)]. Intensity data were collected using ω steps accumulating area detector images spanning at least a hemisphere of reciprocal space. All data were corrected for Lorentz polarization effects. A multiscan absorption correction was applied by using CrysAlisPro. Structures were solved by dual space methods (SHELXT) and refined by full-matrix least-squares against F^2 (SHELXL-2013). Non-hydrogen atoms were refined anisotropically, and hydrogen atoms were refined using a riding model. The structure was modelled using Platons SQUEEZE procedure, and all other calculations were performed using the Crystal Structure interface. Selected crystallographic data are presented in Table S1.

Additional Structural Description. Zn-MUM-1 crystallizes as small yellow prisms and exhibits the space group *C2/c* with an asymmetric unit consisting of one Zn^{2+} cation, one μ_4-odba^{2-} linker and half a μ-bpdh pillar (Figure S1). There is also one full weight solvated DMF molecule in the asymmetric unit. The Zn^{2+} centers in MUM-1 exhibit a square-pyramidal, paddle-wheel geometry with four μ_4-odba^{2-} linkers joining pairs of centers together by occupying the basal

positions and the μ-bpdh linkers coordinating to the apical position, forming inorganic SBUs with the formula $Zn_2N_2O_8C_4$ (Figure S2). Each of the four μ_4-odba^{2-} linkers bound to the SBU coordinate, in the same fashion, to a different SBU. The μ-bpdh pillars run down both the [040] and [140] crystallographic axes. The μ-bpdh pillars are strictly planar, as a function of the symmetry, and the interplanar angles for the aromatic rings in the μ_4-odba^{2-} linkers are 73.35(11)°. The Zn…Zn distance for the paddlewheel SBU is 2.9442(5) Å. There are intra-net π-π stacking interactions between the C13-containing μ_4-odba^{2-} ring and the pyridyl rings, with centroid-centroid distances of 3.67 Å. The free DMF molecules do not hydrogen bond to the framework, as there are no strong donors, but rather sit above the pyridyl ring at a distance of 3.784(8) Å and a pyridyl-DMF interplanar angle of 16.0(7)°. A related XOFYOU structure is isomorphous with Zn-MUM-1 but features a two-fold interpenetration. However, Zn-MUM-1 is a non-interpenetrated MOF. Besides, the SQUEEZE procedure was used for XOFYOU and its R_1 factor is almost double than that of the Zn-MUM-1 dataset (9.16% vs. 4.99%). The authors did not discuss the structure's topology for XOFYOU,[S1] but the geometrical parameters are in close agreement despite the interpenetration of XOFYOU (Table S2). The heterometallic Zn-Cd structures OLOPIQ and OLOPEM are isostructural with XOFYOU, but have Zn/Cd mixtures with Zn^{2+} site occupancies of 0.70 and 0.85, respectively.[S2] The solvent in OLOPIQ was modelled using the SQUEEZE procedure, however the DMF molecule was found in the same place as in Zn-MUM-1 for OLOPEM. Both OLOPIQ and OLOPEM were found to efficiently photocatalyse the degradation of phenols in aqueous solution without the need for auxiliary oxidants like H_2O_2. Both compounds were made, alongside the Cd^{2+} analogue, via mechanochemical synthesis and OLOPIQ was found to be the most efficient catalyst of the three. The mechanism was reliant on

the phenols being able to access the azine groups in the channels, which the smaller internal surface area of the Cd^{2+} analogue prohibited. OLOPIQ was found to be a more efficient catalyst than OLOPEM and the authors reasoning was that it had a higher Cd^{2+} content, whilst maintaining the accessible pore structure.[S2]

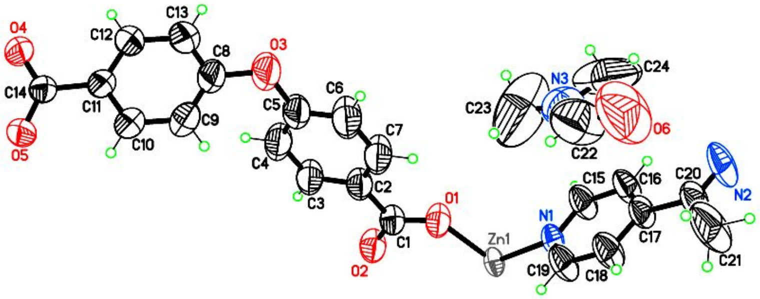

Figure S1. Thermal ellipsoid plot of the asymmetric unit of Zn-MUM-1, with thermal ellipsoids shown at the 50% probability level with atoms labelled with the crystallographic numbering scheme. Hydrogen atoms are shown as spheres of arbitrary radii.

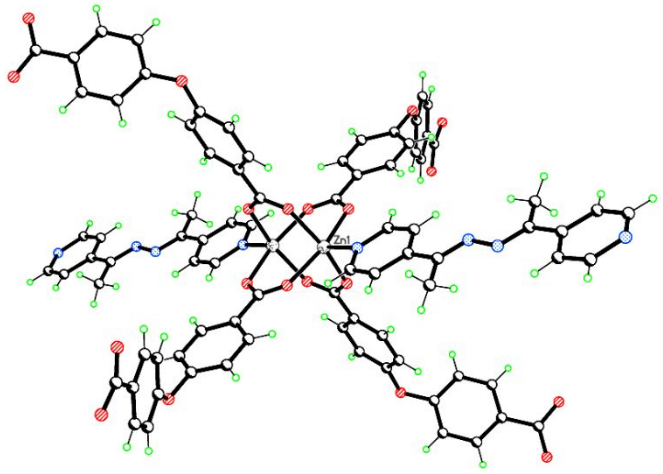

Figure S2. The metal center environment of Zn-MUM-1, showing the paddle-wheel, $Zn_2N_4O_8C_4$ SBU, and all directly coordinated linkers. Solvent molecules were omitted for clarity.

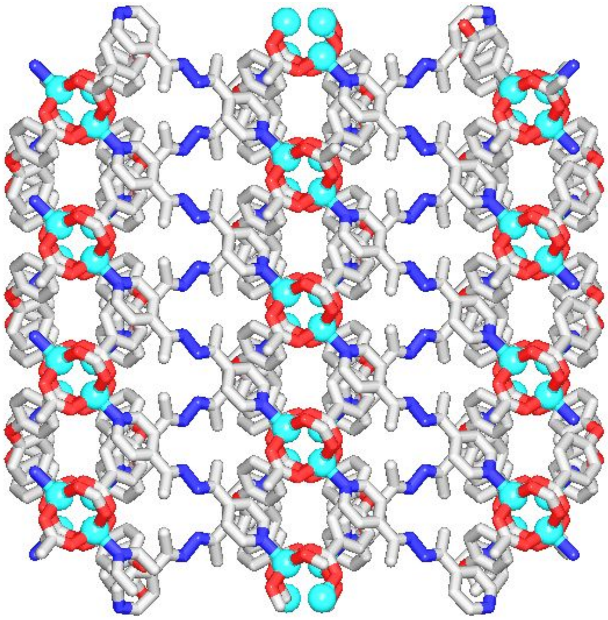

Figure S3. 3D metal-organic framework in Zn-MUM-1. H atoms and DMF solvent molecules were omitted for clarity, color codes: Zn (cyan), O (red), N (blue), C (gray); view along the *c* axis.

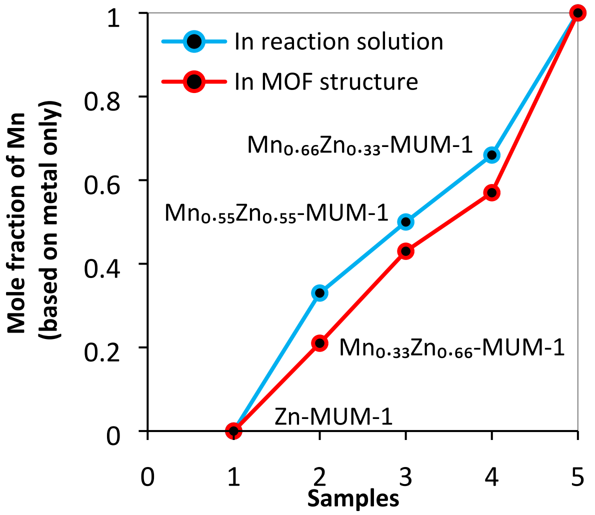

Figure S4. ICP-OES analysis results for Zn and Mn in the prepared samples (dissolved in 0.5 M HCl) and the corresponding reaction solution, wherein the molar ratio of Zn vs. Mn in the reaction solution and Zn-MUM-1 and MnZn-MUM-1 structures is marked for each data point.

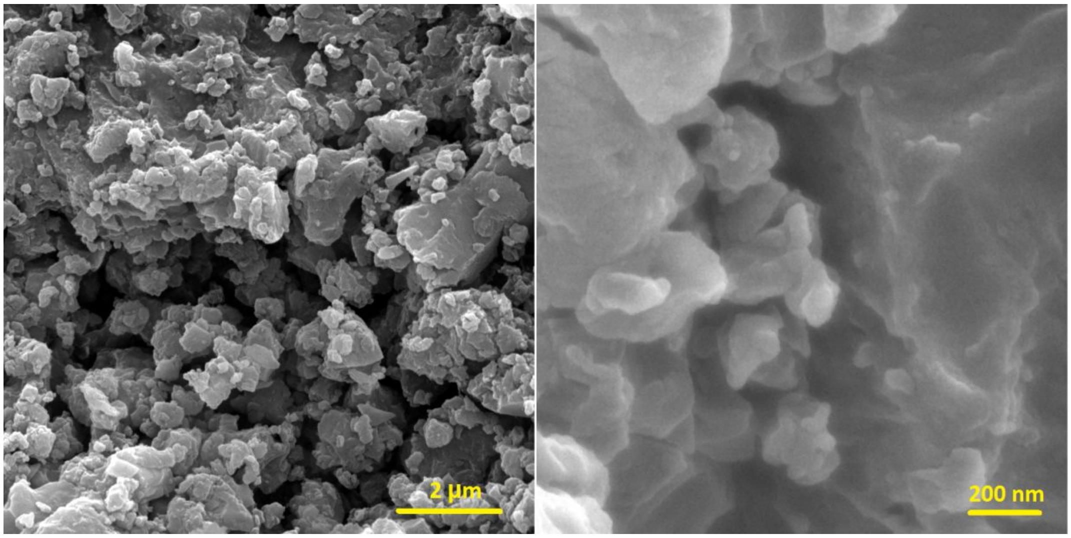

Figure S5. FE-SEM images of $Mn_{0.5}Zn_{0.5}$-MUM-1 at two different resolutions: 2 µm (left) and 200 nm (right).

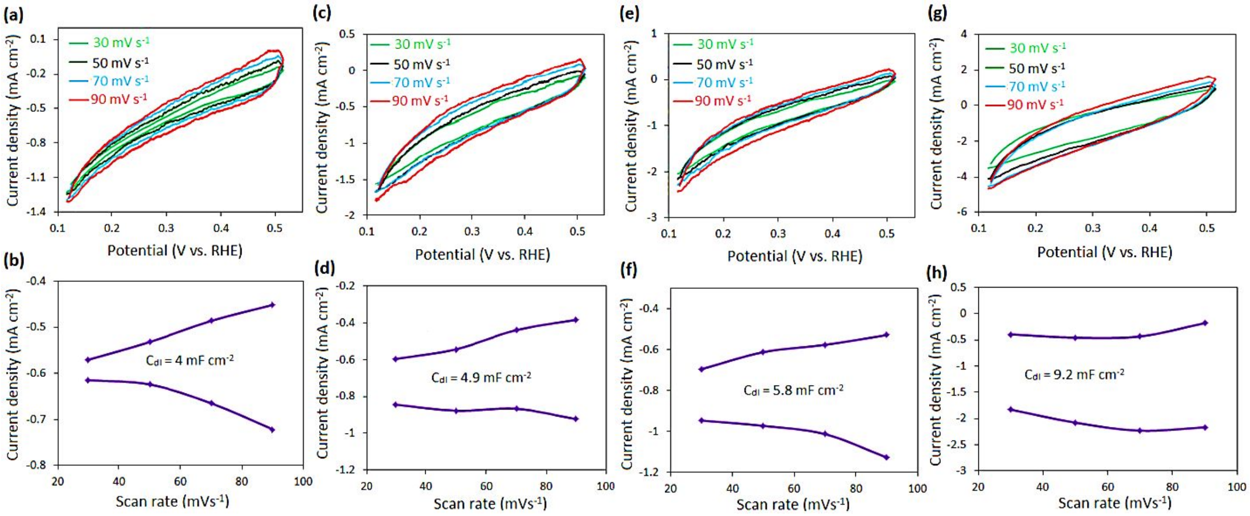

Figure S6. CV curves of Zn-MUM-1/NF (a), $Mn_{0.66}Zn_{0.33}$-MUM-1/NF (c), $Mn_{0.33}Zn_{0.66}$-MUM-1/NF (e), and $Mn_{0.5}Zn_{0.5}$-MUM-1/NF (g) at different scan rates; their related i-scan rate curves are given in (b), (d), (f), and (h), respectively.

Table S1. Crystal data and structure refinement of Zn-MUM-1.

**Identification code**	**Zn-MUM-1**
Empirical formula	$C_{90}H_{74}N_{10}O_{22}Zn_4$
Formula weight	1909.07
Crystal system	monoclinic
Temperature	125 K
Space group	C2/c
Z value	2
F_{000}	1960
Density	1.249 g/cm^3
Crystal size	0.09 × 0.06 × 0.05 mm^3
Cell volume	5076.93(11) Å^3
Unit cell dimensions	a= 27.2863(3) Å
	b= 8.01070(10) Å
	c= 23.5382(3) Å
	ß = 99.3330(10)°
Index ranges	-19 ≤ h ≤ 19
	-20 ≤ k ≤ 20
	-23 ≤ l ≤ 23
Absorption correction	Multi-scan
Absorption coefficient	1.643 mm^{-1}
Reflections collected/unique	27471/ 4817
Max. and min. transmission	0.773 and 0.836
Refinement method	full-matrix least-squares on F^2
Goodness-of-fit on F^2	1.082
Largest diff. Peak, hole	0.646 and -0.482 e$^-$/Å^3
Final R indices (I>2.00s(I))	R1 = 0.0499, wR_2 = 0.1455
R indices (all data)	0.0520

Table S2. Selected bond lengths and angles for Zn-MUM-1 and XOFYOU, showing close agreement.

**Bond length (Å)**	**Zn-MUM-1**	**XOFYOU**	**Bond angles (°)**	**Zn-MUM-1**	**XOFYOU**
Zn1-Zn1	2.9442(5)	2.9374(18)	O1-Zn1-O2	159.45(8)	160.1(2)
Zn1-O1	2.0432(17)	2.045(6)	N1-Zn1-O4	100.05(8)	101.3(3)
Zn1-O2	2.0505(18)	2.055(6)	O1-Zn1-O4	89.30(9)	88.8(3)
Zn1-N1	2.0395(17)	2.033(6)	N1-Zn1-O2	102.95(8)	103.1(3)

Table S3. Parameters obtained from the nitrogen desorption isotherm experiments.

Samples	BET surface area (m^2 g^{-1})	Pore volume (cm^3 g^{-1})	Pore size (Å)
Zn-MUM-1	614	0.28	18.56
$Mn_{0.33}Zn_{0.66}$-MUM-1	585	0.28	18.11
$Mn_{0.5}Zn_{0.5}$-MUM-1	527	0.27	17.08
$Mn_{0.66}Zn_{0.33}$-MUM-1	511	0.25	16.90

Possible Reasons for Lower BET surface Areas of Heterometallic Samples. One of the reasons can include increased internal defects in bimetallic MOFs compared to monometallic ones. Based on Salama et al., who synthesized a series of bimetallic MOFs with different ratios, $Co_{0.5}$–$Mn_{0.5}$-MOF-74 exhibited a larger surface area compared to the other samples ($Co_{0.25}$–$Mn_{0.75}$-MOF-74 and $Co_{0.75}$–$Mn_{0.25}$-MOF-74), which can be due to synergic effect of these two metals.[S3] Particle size enhancement or decrement after the formation of bimetallic or trimetallic MOFs can also alter the surface area. Zhang et al. indicated an increase in the surface area of Co-Ni MOFs compared to monometallic Co MOF due to the decrease in the particle size.[S4] Parida et al. synthesized a bimetallic Ce/Zr MOF and observed a decline in the surface area upon insertion of Ce ions into the framework. The structure remained unchanged after the insertion of Ce ions as it resembled an initial Zr MOF.[S5] These authors and some other groups concluded that this decrease in the surface area of bimetallic MOFs had no impact on the catalytic performance due to the synergic effects of the two metals.[S5-S7] Wang et al. also synthesized NiCo-MOF (190 m^2 g^{-1}), Ni-MOF (191 m^2 g^{-1}), and Co-MOF (261 m^2 g^{-1}).[S8] The surface area of the bimetallic MOFs was smaller than their corresponding monometallic MOFs.[S8] Additionally, Fan et. al. showed that introducing Fe ions to the structure not only resulted in the formation of mixed metal clusters but also altered the medium surrounding the structure which may affect the surface area.[S9] Based on El-Yazeed et al., the surface area and pore volume of bimetallic Cu-Ag MOF were incremented compared to the monometallic MOFs which can be attributed to the formation of new micropores in the bimetallic MOFs.[S10] In summary, several factors can be involved in the decline or increase of the surface area upon the insertion of the second metal into MOF structure and there is a need for further systematic investigations of these aspects.

Supporting References

(S1) Masoomi, M.Y.; Stylianou, K.C.; Morsali, A.; Retailleau, P.; Maspoch, D. Selective CO2 Capture in Metal–Organic Frameworks with Azine-Functionalized Pores Generated by Mechanosynthesis. *Cryst. Growth Des.*, **2014**, *14*, 2092-2096.

(S2) Masoomi, M.Y.; Bagheri, M.; Morsali, A.; Junk, P.C. High photodegradation efficiency of phenol by mixed-metal–organic frameworks *Inorg. Chem. Front*. **2016**, *3*, 944-951.

(S3) Salama, R.S.; Manna, M.A.; Altass, H.M.; Ibrahim, A.A.; Khder, A.E.R.S., Palladium supported on mixed-metal–organic framework (Co–Mn-MOF-74) for efficient catalytic oxidation of CO, *RSC Adv*., **2021**, *11*, 4318−4326.

(S4) Zhang, X.; Luo, J.; Wan, K.; Plessers, D.; Sels, B.; Song, J.; Chen, L.; Zhang, T.; Tang, P.; Morante, J.R.; Arbiol, J.; Fransaer, J. From rational design of a new bimetallic MOF family with tunable linkers to OER catalysts, *J. Mater. Chem. A*, **2019**, *7*, 1616−1628.

(S5) Tripathy, S.P.; Subudhi, S.; Ray, A.; Behera, P.; Bhaumik, A.; Parida, K. Mixed-valence bimetallic Ce/Zr MOF-based nanoarchitecture: A visible-light-active photocatalyst for ciprofloxacin degradation and hydrogen evolution, *Langmuir*, **2022**, *38*, 1766−1780.

(S6) Jin, L.; Liu, H.; Xu, A.; Wu, Y.; Lu, J.; Liu, J.; Xie, S.; Yao, Y.; Dong, L.; Zhang, M.; Kai, S.; Fan, M. Defective UiO-66-NH_2 (Zr/Ce) catalyzes the synthesis of propylene carbonate under mild conditions, *Microporous Mesoporous Mater*., **2021**, *317*, 110997.

(S7) Tripathy, S.P.; Subudhi, S.; Das, S.; Ghosh, M.K.; Das, M.; R. Acharya, Acharya, R.; Parida, K. Hydrolytically stable citrate capped Fe_3O_4@UiO-66-NH_2 MOF: A hetero-structure composite with enhanced activity towards Cr (VI) adsorption and photocatalytic H_2 evolution, *J. Colloid Interface Sci*., **2022**, *606*, 353−366.

(S8) Wang, H.; Li, X.; Lan, X.; Wang, T. Supported ultrafine NiCo bimetallic alloy nanoparticles derived from bimetal-organic frameworks: A highly active catalyst for furfuryl alcohol hydrogenation, *ACS Catal*., **2018**, *8*, 2121–2128.

(S9) Hu, Y.; Zhang, J.; Huo, H.; Wang, Z.; Xu, X.; Yang, Y.; Lin, K.; Fan, R. One-pot synthesis of bimetallic metal–organic frameworks (MOFs) as acid–base bifunctional catalysts for tandem reaction, *Catal. Sci. Technol*., **2020**, *10*, 315–322.

(S10) Abo El-Yazeed, W.S.; Ahmed, A.I. Monometallic and bimetallic Cu–Ag MOF/MCM-41 composites: structural characterization and catalytic activity, *RSC Adv*., **2019**, 9, 18803–18813.
